# The association between diet quality, dietary patterns and depression in adults: a systematic review

**DOI:** 10.1186/1471-244X-13-175

**Published:** 2013-06-27

**Authors:** Shae E Quirk, Lana J Williams, Adrienne O’Neil, Julie A Pasco, Felice N Jacka, Siobhan Housden, Michael Berk, Sharon L Brennan

**Affiliations:** 1 School of Medicine, Deakin University, Geelong, Australia; 2 Department of Psychiatry, The University of Melbourne, Parkville, Australia; 3 School of Public Health and Preventive Medicine, Monash University, Melbourne, Australia; 4 Northwest Academic Centre, Department of Medicine, The University of Melbourne, Sunshine Hospital, 176 Furlong Road, St Albans, Australia; 5 Orygen Youth Health Research Centre, Parkville, Australia; 6 Mental Health Research Institute, Parkville, Australia; 7 Australian Institute for Musculoskeletal Science, The University of Melbourne, 176 Furlong Road, St Albans, Australia

**Keywords:** Depression, Diet, Food habits, Adults, Systematic review

## Abstract

**Background:**

Recent evidence suggests that diet modifies key biological factors associated with the development of depression; however, associations between diet quality and depression are not fully understood. We performed a systematic review to evaluate existing evidence regarding the association between diet quality and depression.

**Method:**

A computer-aided literature search was conducted using Medline, CINAHL, and PsycINFO, January 1965 to October 2011, and a best-evidence analysis performed.

**Results:**

Twenty-five studies from nine countries met eligibility criteria. Our best-evidence analyses found limited evidence to support an association between traditional diets (Mediterranean or Norwegian diets) and depression. We also observed a conflicting level of evidence for associations between (i) a traditional Japanese diet and depression, (ii) a “healthy” diet and depression, (iii) a Western diet and depression, and (iv) individuals with depression and the likelihood of eating a less healthy diet.

**Conclusion:**

To our knowledge, this is the first review to synthesize and critically analyze evidence regarding diet quality, dietary patterns and depression. Further studies are urgently required to elucidate whether a true causal association exists.

## Background

Depressive disorders currently impose a significant health and economic burden in both developed and developing countries. With prevalence estimates ranging between 3.3–21.4% [1], the global burden of depression is now a major public health concern [2]. As such, the identification of modifiable risk factors for depression is an important and pressing research imperative [3].

Recent data have highlighted the importance of the contribution of modifiable lifestyle behaviors such as physical inactivity, smoking, and other lifestyle factors to the development of common mental disorders [4-6]. In addition, the relationship between nutrition and depressive disorders has become of increasing interest in recent years [7,8] in both observational and clinical studies; however, much previous research has focused on the intake of individual nutrients or food groups and their association with depression, or on nutritional supplementation as a treatment strategy in depression.

In this regard, studies have identified associations between the intake of dietary nutrients such as zinc, magnesium, B-group vitamins, culinary fat (such as olive oil), and single food groups such as seafood or fish consumption and decreased risk of depression [8-11]. However, there are important limitations to studying individual nutrients in relation to disease, given the complex combinations and interactions among nutrients in an individual’s daily diet. Diet is a multidimensional exposure and thus it remains difficult to attribute differential disease prevalence or symptomatology to a single nutrient or food group. Moreover, nutrient intake is associated with particular dietary patterns, which may act as confounders in diet-disease associations. As such, dietary patterns are being increasingly examined as predictors of disease outcomes. For example, in a study of middle-aged women participating in the Nurses’ Health Study, a prudent dietary pattern was characterized by higher intakes of vegetables, fruit, legumes, fish, poultry and whole grains, while a western pattern was characterized by higher intakes of red and processed meats, desserts, refined grains and fried foods. These patterns were, in turn, associated with markers of systemic inflammation [12]. Moreover, a western dietary pattern has been shown to increase the risk, and a prudent dietary pattern to reduce the risk, of other inflammatory diseases, for instance, coronary heart disease in both women [13] and men [14]. Salient characteristics of diet may also be captured using a composite measure of dietary intake or dietary quality scores derived from recommended dietary guidelines. For the purpose of this review we define diet quality and dietary patterns as the quality of overall habitual dietary intake, and the pattern of overall habitual dietary intake, respectively, which is consistent with prior research [7,15]. Given the relatively new data available in this field, the aim of this study was to conduct the first systematic review to examine the association between overall diet quality and depression in adults.

## Methods

This systematic review adheres to the guidelines addressed in the preferred reporting items for systematic reviews and meta-analyses (PRISMA) statement 2009 [16] (Additional file 1).

### Eligibility criteria for considering studies for this review

Articles were eligible for inclusion if they: (i) were full-text articles; (ii) comprised cohort, case–control or cross-sectional study designs; (iii) examined associations between self-reported diet quality, defined as the quality of one’s overall habitual food intake ascertained by healthy eating guidelines or *a priori* diet quality score (rather than (1) individual nutrients, (2) individual food items or, (3) individual food groups), or dietary pattern analysis, and depression or depressive symptoms defined by either self-report or the application of diagnostic measurement tools in adults; and (iv) comprised study samples that were population based rather than from acute settings (for example, residents at aged care facilities, in-patients at psychiatric hospitals).

### Criteria for excluding studies from this review

Studies were excluded if they: (i) were published in languages other than English; (ii) utilized animal models; (iii) investigated energy intake as the primary variable of interest or outcome measure; (iv) investigated individual dietary nutrients or single dietary components as the primary variables of interest; (v) investigated malnutrition, including nutritional risk, or disordered eating; (vi) investigated parenteral nutrition as the primary variable of interest; (vii) employed qualitative methodology; (viii) were randomized controlled trials; or (ix) were dissertations. Due to differences in the diagnostic tools used to assess depression in children and/or adolescents compared to adults, we excluded studies that examined diet and depression in populations other than adults.

### Search strategy for identification of studies

A computerized search strategy was implemented using Medline (largest subset of PubMed), CINAHL, and PsycINFO for citations of relevant articles, which were restricted to January 1965 to 31st October 2011. The following medical subject headings (MeSH) were applied: “diet” OR “food habits” AND “depression” OR “depressive disorder” OR “depressive disorder, major”. Keywords were applied to complete the final search strategy: “diet” OR “food habits” OR “dietary” OR “dietary patterns” OR “dietary quality” OR “western diet” or “Mediterranean diet” AND “depression (MeSH)” OR “depressive disorder” OR depressive disorder, major” OR “depression (keyword). Two reviewers confirmed the search strategy (SEQ and SLB) and one reviewer performed the computerized search (SEQ). Complete details of the search strategy can be obtained from the corresponding author.

Reference lists of relevant studies deemed eligible for inclusion were manually searched, and citations were tracked for those publishing in the field of interest (SEQ). Two reviewers (SEQ and SLB) confirmed the selection of articles based on readings of the full text article. Where the eligibility of studies was ambiguous, two reviewers held discussions to reach consensus (SEQ and SLB). Where consensus could not be achieved, a third reviewer was consulted (LJW).

### Methodological quality of included manuscripts

Two reviewers (SEQ and SLB) independently assessed the quality of the studies by scoring them using an adaptation of Lievense et al.’s scoring system [17,18] (Table 1). Each of the 14 criteria items were scored as follows: positive (1), negative (0), or unclear (?) with 100% representing a maximum possible score. A third reviewer (LJW) provided a final judgment where the reviewers’ agreement could not be reconciled. Studies were defined as high quality if the total quality score for all quality scores were above the mean. The optimal design was considered to be cohort studies, followed by case–control studies and, finally, cross-sectional study designs.

**Table 1:** **Criteria list for the assessment of study quality, modified from Lievense et al**[15,16]

**Item**	**Criteria**	**C/CC/CS**^**†**^
*Study population*		
1	Selection at uniform point	C/CC/CS
2	Cases and controls drawn from the same population	CC
3	Participation rate >80% for cases/cohort	C/CC
4	Participation rate >80% for controls	CC
*Assessment of risk factor*		
5	Exposure assessment blinded	C/CC/CS
6	Exposure measured identically for cases and controls	CC
7	Exposure assessed according to validated measures	C/CC/CS
*Assessment of outcome*		
8	Outcome assessed identically in studied population	C/CC/CS
9	Outcome reproducibly	C/CC/CS
10	Outcome assessed according to validated measures	C/CC/CS
*Study design*		
11	Prospective design used	C/CC
12	Follow-up time ≥12 months	C
13	Withdrawals <20%	C
*Analysis and data presentation*		
14	Appropriate analysis techniques used	C/CC/CS
15	Adjusted for at least age, and gender	C/CC/CS

^**†**^C = applicable to cohort studies, CC = applicable to case-control studies, CS = applicable to cross-sectional.

### Data analysis

Our decision not to proceed with a meta-analysis of the data from reviewed studies was determined *a priori.* Given the current work in this field of enquiry being undertaken by the authors, our group had an appreciation of the inherent heterogeneity of these studies, largely related to measurement of diet and assessment of depression. Our “best-evidence synthesis” consisted of five levels of evidence ranging from strong evidence (1), moderate evidence (2), limited evidence (3), conflicting evidence (4), to no evidence (5), which reflected the type of study design used (Table 2).

**Table 2:** **Criteria for ascertainment of evidence level for best-evidence synthesis, adapted from Lievense et al**[15,16]

**Level of evidence**	**Criteria for inclusion in best evidence synthesis**
Strong evidence	Generally consistent findings in:
	Multiple high-quality cohort studies
Moderate evidence	Generally consistent findings in:
	One high-quality cohort study and >2 high quality case-control studies
Limited evidence	Generally consistent findings in:
	Single cohort study
	One or two case-control studies or
	Multiple cross-sectional studies
Conflicting evidence	Inconsistent findings in <75% of the trials
No evidence	No studies could be found

## Results

### Identification and selection of the included manuscripts

**Figure 1:**
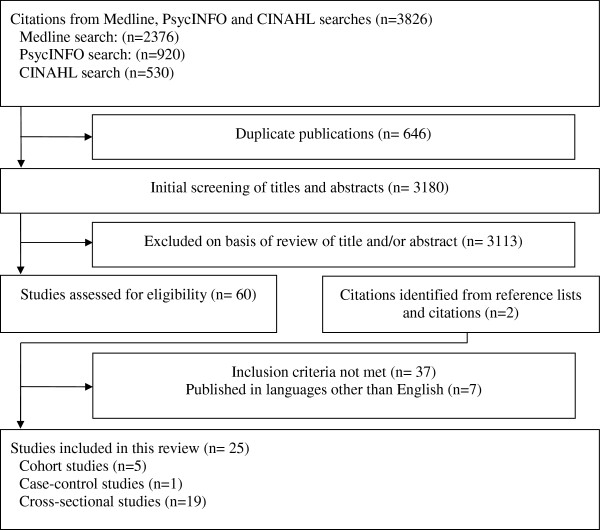
Summary of systematic search presented as an adapted CONSORT diagram.

Utilizing Medline, CINAHL, and PsycINFO databases, the computer-assisted search generated a total of 3,826 articles, of which 646 articles were duplicates. The title and/or abstracts of the remaining 3,180 articles were screened for eligibility, of which 3,113 were excluded due to failing to meet preliminary eligibility criteria, and seven were excluded as they were printed only in languages other than English. A further 37 articles were excluded failing to meet eligibility criteria, based on a concise reading of the full articles. One further article [19] was identified when searching the reference lists of articles meeting inclusion criteria, and the e-pub of another article [20] was identified when tracking authors publishing in the field of interest. The final number of studies to be included in the review was 25 (Figure 1).

Studies most frequently failed to meet eligibility criteria for inclusion for the following reasons (i) examined individual nutrients or supplements, rather than overall dietary quality, (ii) examined fat intake rather than overall dietary quality, (iii) examined individual food items or food groups including fish intake or meat intake, (iv) examined malnutrition or nutritional risk rather than overall dietary quality, or (v) did not measure depression or depressive symptoms adequately.

### Methodological quality of included manuscripts

The two reviewers (SEQ and SLB) scored a potential 253 criteria over the 25 studies, resulting in an inter-rater reliability of 84%. The majority of the discrepancies were resolved in one consensus meeting; however, the third reviewer (LJW) provided final judgment on 1.9% of the items.

The quality scores ranged from 55.6% [19] to 100% [7,20-23] of the maximum obtainable score for each of the study designs. Only high-quality studies (as determined by methodological assessment quality score above the mean of 87.73%) were included in the best evidence synthesis. However, all of the reviewed studies scored above the 50% of possible methodological quality. High levels of heterogeneity remained in the 18 studies determined as high quality [7,8,15,20-34], thus statistical pooling of the extracted data was not feasible. Therefore, a best evidence synthesis was performed to assess the associations, as previously published in other fields [17,35].

### Description of the studies

An overview of the reviewed studies (n = 25) is presented in Table 3. Five of the eligible studies were a cohort design [15,20,21,23,36], one was a case–control design [37] and the remaining 19 studies were cross-sectional [7,8,19,22,24-34,38-41]. The majority of studies were published from 2009 onwards (n = 22, 84.6%), with precise publication years being; 2002 [26], 2007 [30], 2008 [25], 2009 [19,21,22,24,28,29,32,36-38], 2010 [7,33,34] and 2011 [8,15,20,23,27,31,39-41]. Eleven studies were undertaken in United States of America (USA) [19,26,29,31-34,39-42], three were conducted in Japan [22,23,27], two were undertaken in Spain [20,36]. Remaining studies were conducted in the United Kingdom (UK) [21] Greece [15], Korea [37] China [30], France [25], Australia [7], Norway [8], Mediterranean Islands [28] and Europe (a combination of populations from Germany, Poland and Bulgaria) [38]. Sample sizes ranged from a cross-sectional study with 50 participants [41] to a cohort study with 10,094 participants [36] with the total number of participants examined by this review summing 53,770. Age ranges varied from 20 years in three cross-sectional studies [7,33], up to 100 years in another cross-sectional study [28]. Nine of the reviewed studies examined populations comprising only females [7,15,19,23,26,31,37,40],[41] whilst one study comprised only of male populations [39], and the remaining 15 studies were mixed with regards to sex.

**Table 3:** Study characteristics of eligible studies included in this review, grouped by study design, year of publication, and author

**Author, country of study, year**	**n = subjects (%females)**	**Age, years; Mean (±SD) or range, yr**	**Population description**	**Dietary assessment**		**Depression assessment**		**Quality score%**
				**Tool**	**Type**	**Tool**	**Cut-off**	
*Cohort*								
Akbaraly et al., UK, 2009 [21]	3486 (26.2)	55.6 (*), 35–55	White European participants in the Whitehall II study with diet data at 1997–9, and depression data at 2002–4	FFQ, validated, 127 items	(i) Whole food	CES-D	>15	100
					(ii) Processed food			
Sanchez-Villegas et al., Spain, 2009 [36]	10,094 (% in categories of adherence to Med. diet; 0–2: 59.9 3: 61.4 4: 58.0 5: 57.4 6–9: 56.0)	Age in categories of adherence to Med. diet; 0–2: 33.3 (9.8) 3: 35.7 (10.7) 4: 36.8 (11.3) 5: 38.0 (11.6) 6–9: 41.3 (12.1)	SUN Spanish cohort of former students of University of Navarra, registered professionals from some Spanish provinces and other university graduates	FFQ, validated, 136 items	Mediterranean diet	Self-reported question	–	83.3
Chatzi et al., Greece, 2011 [15]	529 (100)	*	Prospective mother-child cohort, recruitment mid-pregnancy, follow up 8–10 weeks post-partum	FFQ, validated for this particular cohort, 250 items	(i) Western pattern	EPDS	≥13	91.7
					(ii) Healthy pattern			
Okubu et al., Japan, 2011 [23]	865 (100)	29.9 (4.0)	Pregnant females enrolled in the Osaka Maternal and Child Health Study, recruited 2001–3, follow up 2–9 months post-partum	DHQ, validated, 145 items	(i) Healthy diet	EPDS	≥9	100
					(ii) Western diet			
					(iii) Japanese diet			
Sanchez-Villegas et al., Spain, 2011 [20]	8,964 (*)	*	SUN Spanish cohort of former students of University of Navarra, registered professionals from some Spanish provinces and other university graduates	FFQ, validated, 136 items, 2 × 24 hour diet recalls	(i) Fast food	Self-reported question	–	100
					(ii) Commercial baked goods			
*Case–control*								
Park et al., Korea, 2010 [37]	130 (100)	Cases: 20.6 (0.2)	Korean female College students residing in Incheon area, recruited 2009	Independently constructed self-reported dietary habits questionnaire 16 items	(i) Dietary pattern of meat, fish, eggs, beans more than twice a day	CES-D	≥16	84.6
		Control: 20.5 (0.2)						
					(ii) Total dietary habits score			
*Cross-sectional*								
Tangney et al., USA, 2002 [26]	117 (100)	61.5 (*)	Female breast cancer patients of urban teaching hospital, cancer diagnosis 0.5–5 years prior to 1997	HHHQ transcribed to modified Block FFQ, HEI	Diet quality ascertained by HEI score	CES-D	≥16	88.9
Liu et al., China, 2007 [30]	2,579 (42.1)	20.4 (*)	College students over 7 cities in China, recruited 2003–4	Independently constructed FFQ specifically for study	(i) Ready to eat food	CES-D, adapted to use 3 items	–	88.9
					(ii) Snack food			
					(iii) Fast food			
Samieri et al., France, 2008 [25]	1,724 (62.5)	65+	Community-dwelling residents of Bordeaux, France, enrolled in Three-City study, recruited 2001–2	FFQ, 24 hour diet recall	(i) Biscuits and snacking	CES-D, hybrid analyses	–	88.9
					(ii) Healthy diet			
					(iii) Charcuterie, starchy foods (women) ^ψ^			
					(iv) Pizza, sandwich (women)			
Jeffery et al., USA, 2009 [19]	4,655 (100)	52.4 (6.6)	Telephone survey of females enrolled in the Group Health Cooperative who had previously completed survey regarding breast cancer risk	Independently constructed FFQ, 39 items	(i) High calorie sweet diet	PHQ	≥10	55.6
					(ii) High calorie non-sweet diet			
					(iii) Low calorie diet			
Beydoun et al., USA, 2009 [32]	(i) 1789 (56.1)	(i) 30–64	Two subsamples of HANDLS, recruited from initial recruitment phase in 2004; sample (ii) also had information regarding bone quality	USDA,AMPM 2 × 24 hour diet recall, validated, 2005 HEI	Diet quality ascertained by HEI	CES-D	≥16, and ≥20	88.9
		(ii) 30–64						
	(ii) 1583 (56.5)							
Mikolajczyk et al., Europe, 2009 [38]	Germany: 696 (56.6)	20.6 (2.3) (Combined)	First Year College students, subsample of participants enrolled in Cross National Student Health Survey, recruited 2005	FFQ, 12 items	Fast food	M-BDI	≥35	77.8
	Poland: 489 (71.8)							
	Bulgaria: 654 (68.7)							
Pagoto et al., USA, 2009 [24]	210 (78.4)	51.8 (11.2)	Residents of Lawrence, Massachusetts, enrolled in Lawrence Diabetes Prevention Project, 2004–7	3 × 24 hour diet recalls	Alternate HEI	CES-D	≥16	88.9
Beydoun et al., USA, 2010 [34]	1,681 (56.3)	Males: 47.9 (9.3)	Subsample of HANDLS, recruited from initial recruitment phase 2004–8	USDA, AMPM, validated, 2× 24 hour recall, 2005 HEI	Diet quality ascertained by HEI score	CES-D	≥16	88.9
		Females: 47.9 (9.2)						
Beydoun and Wang, USA, 2010 [33]	2,217 (50.3)	20–39	Subsample of NHANES, pooled for periods 1999–2000, 2001–2, 2003–4	USDA, AMPM, validated, 2× 24 hour recall, 2005 HEI	Diet quality ascertained by HEI score	CIDI	<curve AUC = 0.83	88.9
Jacka et al., Australia, 2010 [7]	1,046 (100)	20–93	Females enrolled in Geelong Osteoporosis Study, recruited 1994–7	FFQ, validated, 74 items	(i) Western diet	SCID-I/NP	–	100
					(ii) Traditional diet			
					(iii) ‘Modern’ diet			
Kuczmarski et al., USA, 2010 [29]	1,118 (55.7)	48.4 (0.3)	Subsample of HANDLS, urban population, recruited from initial recruitment phase 2004–8	USDA 2005 HEI, 2 x dietary recalls	Total diet quality	CES-D	≥16	88.9
Mamplekou et al., Mediterranean Islands, 2010 [28]	1,190 (53.5)	65–100	Randomly recruited, population-based sample of elderly individuals residing in the Republic of Cyprus, and the islands of Mitilini, Samothraki, Cephanlonia, Crete, Corfu, Lemnos and Zakynthos	FFQ, validated, MedDietScore	Mediterranean diet	GDS	>10	88.9
Nanri et al., Japan, 2010 [22]	521 (40.7)	21–67	Employees of two municipal offices in Northeastern Kyushu, Japan, who attended a periodic health examination, recruited 2006	BDHQ, validated, 65 items, Principle component analysis	(i) Healthy Japanese diet pattern	CES-D	≥16	100
					(ii) Animal food pattern			
					(iii) Westernized breakfast pattern			
Aihara et al., Japan, 2011 [27]	833 (56.5)	Males: 76.1 (5.0)	Random recruitment from rosters of community associations of Odawara, Japan	Independently constructed, self-reported dietary habits, single question “ Do you eat well-balanced meals (i.e., intake of a variety of food with staple food, as well as main and side dishes)?	Well balanced diet	GDS-5	≥2	88.9
		Females: 74.9 (5.5)						
Castellanos et al., USA, 2011 [39]	75 (0)	29.6 (8.2)	Latino males residing in Mississippi, convenience sample	The Block fat and fruit and vegetable screening tool for	(i) Fruit and vegetable	CES-D	≥16	77.8
				Mexican Americans, validated	(ii) Fat intake			
Crawford et al., USA, 2011 [40]	626 (100)	45–54	Females enrolled in the Midlife Health Study, recruited 2002–4	Single question “How often did you eat foods from the following restaurants during the past year?”	Fast food frequency	CES-D	≥16	77.8
Fowles, Timmerman et al. USA, 2011 [41]	50 (100)	24.0 (*)	Low-income females in first trimester of pregnancy, identified as uninsured or underinsured by Texas-based insurance records, recruited 2009	DQI-P, 3 × 24 hour diet recall	Fast food frequency	EPDS	≥10	77.8
Fowles, Bryant et al. USA, 2011 [31]	118 (100)	25.3 (5.3)	Low-income females in first trimester of pregnancy, identified as uninsured or underinsured by Texas-based insurance records, recruited 2009-10	DQI-P, 3 × 24 hour diet recall	Total diet quality	EPDS	≥10	88.9
Jacka et al., Norway, 2011 [8]	5,731 (56.8)	46–49 (n = 2,957)	Subsample of Hordaland Health Study, participants from four communities, born in years 1925–7 or 1950–1	FFQ, validated, 169 items	(i) Healthy diet	HADS-D	≥8	88.9
		70–74 (n = 2,774)			(ii) Western diet			
					(iii) Norwegian diet			
					(iv) Diet quality score			

* Data not provided.

Abbreviations: *FFQ* Food Frequency Questionnaire, *USDA* United States Department of Agriculture, *AMPM* Automated Multiple Pass Method, *HEI* Healthy Eating Index, *CES-D* Centre for Epidemiological Studies Depression, *GDS* Geriatric Depression Scale, *EPDS* Edinburgh Postnatal Depression Scale, *DHQ* Diet History Questionnaire, *BDHQ* Brief Dietary History Questionnaire, *PHQ* Patient Health Questionnaire, *CIDI* Composite International Diagnostic Interview (Version 2.1), *SCID-I/NP* Structured Clinical Interview for DSM-IV-TR Research Version, Non-Patient Edition, *HADS-D* Hospital Anxiety and Depression Scale for depression, *M-BDI* Modified Beck Depression Inventory, *HANDLS* Healthy Aging in Neighborhoods of Diversity across the Life Span, *SUN* Seguimiento Universidad de Navarra, *DQI-P* Dietary Quality Index-Pregnancy.

^ψ^ The analysis undertaken for male participants by Samieri et al. [25] was based on a food pattern of meat consumption and thus ineligible for inclusion.

Whilst some of the studies were population-based, there were also a range of specific population groups including: a sample of white European office workers from the Whitehall II study [21], municipal office employees attending a periodic health examination [22]; university graduates and registered professionals from the Seguimiento Universidad de Navarra/University of Navarra follow-up (SUN) cohort [20,36]; College students [30,37,38]; pregnant women [15,23,31,41], and women with breast cancer [26].

A wide variety of tools, and combinations of the tools, were employed to ascertain habitual dietary intakes. These included: (i) a validated food frequency questionnaire (FFQ) [7,8,15,20,21,26,28,36] (ii) independently constructed or modified versions of a validated FFQ [19,25,30,38,40], (iii) validated Brief Diet History Questionnaire [22] (iv) USDA Automated Multiple Pass Method (AMPM) [32-34] (v) other dietary recall methods [20,24,25,29,31,36,41], (vi) self-reported questionnaires or a single dietary habits question [27,37,40] and versions of the Block FFQ [26,39]. Methods for determining diet quality also varied and included: validated Diet Quality Scores, including (vii) United States Department of Agriculture (USDA), (viii) Healthy Eating Index (HEI) or Alternative HEI [26,29,32-34,42], (ix) those based on Australian National Dietary Guidelines [7], (x) the Dietary Quality Index-Pregnancy (DQI-P) [31], (xi) Diet Health Questionnaire (DHQ) [23]; two different validated methods to measure Mediterranean diets [28,36] and various other non-validated methods for assessing diet.

Similarly, a range of different methods were used to identify cases of depression, the most common being the Center for Epidemiological Studies Depression Scale (CES-D) to identify symptomatology, whilst two studies utilized psychiatric diagnostic interviews [7,33]. Another two utilized previous physician-made diagnosis of clinical depression and/or habitual use of antidepressant medications [20,36].

### Study groupings

Due to the substantial heterogeneity of the study designs, particularly in relation to measures of diet quality, subjective decisions were required regarding the way studies were grouped. As such, studies addressing dietary constructs clearly designed to assess the habitual intake of foods known to be healthy were grouped. Similarly, ‘western’ dietary patterns and dietary constructs assessing the intake of foods of lower dietary quality, including takeaway foods, processed foods and those with high sugar and/or fat content, were grouped. Finally, culturally specific ‘traditional’ dietary patterns were grouped, which included Japanese, Norwegian and Mediterranean.

### Findings of the studies

Results of the 25 reviewed studies are presented in Tables 4, 5, 6 and 7, according to the exposure of interest. Where possible, results are presented in the form of odds ratio (OR) with 95% confidence intervals (95% CI), or beta coefficient and standard error (SE) or 95% CI; *p* values are provided where available.

**Table 4:** Summary of associations between traditional dietary patterns and depression, presented by year of publication, and author

**Author, country, year**	**Type of diet**	**Adjusted for confounders**	**Results (G = group, T = tertile, C = category, Q = quartile,)**	**p for trend**	**Summary of associations**
*Cohort*					
Sanchez-Villegas et al., Spain, 2009 [36]	Mediterranean	Age, sex, smoking, BMI, physical activity, energy intake, employment	C1: Referent	**<0.001**	Increased adherence to Mediterranean diet associated with reduced odds of self-reported depression
			C2: 0.74 (0.57, 0.98)		
			C3: 0.66 (0.50, 0.86)		
			C4: 0.49 (0.36, 0.67)		
			C5: 0.58 (0.44, 0.77)		
Sanchez-Villegas et al., Spain, 2009 [36]	Mediterranean	Age, sex, smoking, BMI, physical activity, energy intake, employment, excluding participants with early depression	C1: Referent	**<0.001**	Increased adherence to Mediterranean diet associated with reduced odds of self-reported depression
			C2: 0.73 (0.50, 1.06)		
			C3: 0.56 (0.38, 0.83)		
			C4: 0.42 (0.27, 0.66)		
			C5: 0.50 (0.33, 0.74)		
Sanchez-Villegas et al., Spain, 2009 [36]	Mediterranean	Age, sex, smoking, BMI, physical activity, energy intake, employment, excluding participants using antidepressant medication during follow up without physician diagnosis	C1: Referent	**0.007**	Increased adherence to Mediterranean diet associated with reduced odds of self-reported depression
			C2: 0.79 (0.57, 1.09)		
			C3: 0.67 (0.48, 0.93)		
			C4: 0.56 (0.39, 0.80)		
			C5: 0.69 (0.50, 0.96)		
Okubu et al., Japan, 2011 [23]	Japanese	Age, gestation, parity, smoking, change in diet in preceding month, family structure, occupation, family income, education, season, BMI, time of delivery, medical problems during pregnancy, sex and birth weight of baby	Q1: Referent	0.59	No association
			Q2: 0.56 (0.30, 1.02)		
			Q3: 1.14 (0.66, 1.96)		
			Q4: 0.96 (0.56, 1.64)		
*Cross-sectional*					
Mamplekou, Mediterranean Islands, 2010 [28]	Mediterranean	Age, sex, BMI, living alone, financial status, physical activity, smoking, co-morbidities, education, alcohol, retired, urban/rural area	G1: 1.00 (ref)	NS*	No association
			G2: 1.03 (0.98–1.09)		
Nanri et al., Japan, 2010 [22]	Japanese	Age, sex, workplace	T1: Referent	**<0.001**	Increased adherence to Japanese diet associated with reduced odds of depressive symptoms
			T2: 0.90 (0.57, 1.41)		
			T3: 0.39 (0.23, 0.67)		
Nanri et al., Japan, 2010 [22]	Japanese	Age, sex, workplace, marital status, BMI, job position, physical activity, smoking, co-morbidities, total energy intake	T1: Referent	**0.006**	Increased adherence to Japanese diet associated with reduced odds of depressive symptoms
			T2: 0.99 (0.62, 1.59)		
			T3: 0.44 (0.25, 0.78)		
Jacka et al., Norway, 2011 [8]	Norwegian	Age, income, education, physical activity, smoking, alcohol, energy consumption	Males:		Increased adherence to Norwegian diet associated with reduced odds of depressive symptoms for males
			C1: Referent		
			C2: 0.77 (0.61, 0.96)	**0.02**	
			Females:		No association for females
			C1: Referent		
			C2: 0.99 (0.76, 1.29)	0.51	

* Data not provided, *NS* not significant.

Results presented as Odds Ratio (OR) or Hazards Ratio (HR) and (95% CI), except where indicated by superscripts: ^†^beta regression coefficients (± SE), or ^α^ mean (±SE).

**Table 5:** Summary of associations between a healthy dietary pattern and depression, presented by year of publication

**Author, country, year**	**Type of diet**	**Adjusted for confounders**	**Results (T = tertile, Q = quartile, C = category)**	**p for trend**	**Summary of associations**
*Cohort*					
Akbaraly et al., UK, 2009 [21]	Whole food dietary pattern	Age, gender, energy intake	T1: Referent		Increased adherence to whole food diet associated with reduced odds of depressive symptoms
			T2: 0.62 (0.48, 0.79)	**0.0002**	
			T3: 0.64 (0.49, 0.83)	**0.001**	
Akbaraly et al., UK, 2009 [21]	Whole food dietary pattern	Age, gender, energy intake, marital status, employment, education, physical activity, smoking	T1: Referent		Increased adherence to whole food diet associated with reduced odds of depressive symptoms
			T2: 0.68 (0.52, 0.89)	**0.004**	
			T3: 0.74 (0.56, 0.98)	**0.03**	
Akbaraly et al., UK, 2009 [21]	Whole food dietary pattern	Age, gender, energy intake, marital status, employment, education, physical activity, smoking, co-morbidities, use of anti-depressant drugs, cognitive functioning	T1: Referent		Increased adherence to whole food diet associated with reduced odds of depressive symptoms
			T2: 0.71 (0.54, 0.92)	**0.01**	
			T3: 0.74 (0.56, 0.99)	**0.04**	
Akbaraly et al., UK, 2009 [21]	Whole food dietary pattern	Prior depression, age, gender, energy intake	T1: Referent		Increased adherence to whole food diet associated with reduced odds of depressive symptoms
			T2: 0.63 (0.46, 0.87)	**0.005**	
			T3: 0.66 (0.47, 0.92)	**0.01**	
Akbaraly et al., UK, 2009 [21]	Whole food dietary pattern	Prior depression, age, gender, energy intake, marital status, employment, education, physical activity, smoking	T1: Referent		Increased adherence to whole food diet associated with reduced odds of depressive symptoms (non-linear)
			T2: 0.70 (0.50, 0.96)	**0.03**	
			T3: 0.74 (0.52, 1.04)	0.08	
Akbaraly et al., UK, 2009 [21]	Whole food dietary pattern	Prior depression, age, gender, energy intake, marital status, employment, education, physical activity, smoking, co-morbidities, use of anti-depressant drugs, cognitive functioning	T1: Referent		Increased adherence to whole food diet associated with reduced odds of depressive symptoms (non-linear)
			T2: 0.68 (0.50, 0.94)	**0.02**	
			T3: 0.73 (0.51, 1.02)	0.07	
Chatzi et al., Greece, 2011 [15]	Healthy diet	Age, education, parity, house tenure, depression during previous pregnancies, total energy intake during pregnancy	(Outcome: EPDS)	**0.02**	Increased adherence to healthy diet associated with lower mean depressive symptom scores
			T1: Referent		
			T2:–1.13 (−2.25, 0.00)		
			T3:–1.75 (−3.22,–0.28)		
Chatzi et al., Greece, 2011 [15]	Healthy diet	Age, education, parity, house tenure, depression during previous pregnancies, total energy intake during pregnancy	(Outcome: symptoms)	**0.04**	Increased adherence to healthy diet associated with lower mean depressive symptom scores
			T1: Referent		
			T2: 0.52 (0.30, 0.92)		
			T3: 0.51 (0.25, 1.05)		
Okubu et al., Japan, 2011 [23]	Healthy diet	Age, gestation, parity, smoking, change in diet in preceding month, family structure, occupation, family income, education, season, BMI, time of delivery, medical problems during pregnancy, sex and birth weight of baby	Q1: Referent	0.72	No association
			Q2: 0.82 (0.46, 1.47)		
			Q3: 1.49 (0.86, 2.60)		
			Q4: 0.94 (0.52, 1.69)		
*Case–control*					
Park et al., Korea, 2010 [37]	Total diet quality	Matched for age, sex	Cases: 47.2 ± 0.9	**<0.01**	Increased adherence to healthier total diet associated with lower mean depressive symptom scores
			Controls: 51.3 ± 0.9 ^α^		
Park et al., Korea, 2010 [37]	Meat, fish, eggs, beans < twice per day	Matched for age, sex	Cases: 2.9 ± 0.1	**<0.05**	Increased adherence to diet based on meat, fish, eggs, and bean associated with lower mean depressive symptom scores
			Controls: 3.3 ± 0.1 ^α^		
*Cross-sectional*					
Tangney et al., USA, 2002 [26]	Healthy	Age, BMI, tumor characteristics (stage, node, estrogen receptor), time since breast cancer diagnoses	*	**0.0003**	Increased adherence to healthy diet associated with lower mean depressive symptom scores
Samieri et al., France, 2008 [25]	Healthy	Age, education, income, marital status	Males: −0.12 (−0.31, 0.07) ^**†**^	0.21	No association
			Females: −0.16 (−0.33, 0.007) ^**†**^	0.06	No association
Jeffery et al., USA, 2009 [19]	Low calorie	BMI, energy intake	−0.027 (*)^†^	**<0.001**	Increased adherence to low calorie diet associated with reduced odds of depressive symptoms
Beydoun et al., USA, 2010 [34]	Healthy overall	Age, ethnicity, marital status, education, poverty status, smoking, illicit drug use, BMI	Males: −0.035 (0.025) ^†^	NS*	No association
			Females: −0.070 (0.023) ^†^	**<0.05**	Increased adherence to healthy overall diet associated with reduced odds of depressive symptoms for females
Jacka et al., Australia, 2010 [7]	‘Traditional’ (healthy) dietary pattern	Age, socioeconomic status, education, physical activity, smoking, alcohol energy intake	C1: Referent	**<0.05**	Increased adherence to a traditional diet (vegetables, fruit, meat, fish, wholegrain foods) with reduced odds of depression
			C2: 0.65 (0.43, 0.98)		
Jacka et al., Australia, 2010 [7]	Diet quality score	Age, socioeconomic status, education, physical activity, smoking, alcohol, energy intake	C1: Referent	NS*	No association
			C2: 0.85 (0.65, 1.13)		
Jacka et al., Australia, 2010 [7]	‘Modern’ dietary pattern	Age, socioeconomic status, education, physical activity, smoking, alcohol energy intake	C1: Referent	NS*	No association
			C2: 1.29 (0.96, 1.73)		
Kuczmarski et al., USA, 2010 [29]	Healthy diet quality	Sex, education, income, race	*	**<0.0001**	Increased adherence to healthy diet associated with reduced odds of depressive symptoms
Aihara et al., Japan, 2011 [27]	Well-balanced meals	Age, prior depression, illness, cognitive difficulties, gender	Males:	**<0.05**	Increased adherence to eating well-balanced meals associated with reduced odds of depressive symptoms
			C1: Referent		
			C2: 2.75 (1.25, 6.05)		
			Females:	**<0.01**	
			C1: Referent		
			C2: 2.37(1.27, 4.43)		
Fowles, Bryant et al., USA, 2011 [31]	Total diet quality	Age, education, social support, eating habits	−0.293 (*)^†ψ^	**<0.01**	Healthier total diet quality associated with lower mean depressive symptoms
Jacka et al., Norway, 2011 [8]	Healthy dietary pattern	Age, income, education, physical activity, smoking, alcohol, energy consumption	Males:		
			C1: Referent		
			C2: 1.02 (0.87, 1.19)	0.92	No association
			Females:		
			C1: Referent		
			C2: 0.68 (0.57, 0.87)	**<0.001**	Increased adherence to healthy diet associated with reduced odds of depressive symptoms for females
Jacka et al., Norway, 2011 [8]	Diet quality score	Age, income, education, physical activity, smoking, alcohol, energy consumption	Males: OR (95% CI) per SD increase: 0.83 (0.70, 0.99)	**0.034**	Increased adherence to healthy (total) diet associated with reduced odds of depressive symptoms for males and females
			Females: OR (95% CI) per SD increase: 0.71 (0.59, 0.84)	**<0.001**	

* Data not provided. ^ψ^ Outcome was defined by the combination of depression and stress scores. Results presented as Odds Ratio (OR) or Hazards Ratio (HR) and (95%CI), except where indicated by superscripts: ^†^beta regression coefficients (± SE), or ^α^ mean (±SE).

**Table 6:** Summary of associations between Western/unhealthy dietary intakes and depression, presented by year of publication, and author

**Author, country, year**	**Type of diet**	**Adjusted for confounders**	**Results (C = category, T = tertile, Q = quartile)**	**p for trend**	**Summary of associations**
*Cohort*					
Akbaraly et al., UK, 2009 [21]	Processed food dietary pattern	Age, gender, energy intake	T1: Referent		Increased consumption of processed foods associated with increased odds of depressive symptoms
			T2: 1.28 (0.97, 1.69)	0.08	
			T3: 1.75 (1.25, 2.45)	**0.001**	
Akbaraly et al., UK, 2009 [21]	Processed food dietary pattern	Age, gender, energy intake, marital status, employment, education, physical activity, smoking	T1: Referent		Increased consumption of processed foods associated with increased odds of depressive symptoms
			T2: 1.22 (0.92, 1.62)	0.17	
			T3: 1.58 (1.12, 2.23)	**0.009**	
Akbaraly et al., UK, 2009 [21]	Processed food dietary pattern	Age, gender, energy intake, marital status, employment, education, physical activity, smoking, co-morbidities, use of anti-depressant drugs, cognitive functioning	T1: Referent		Increased consumption of processed foods associated with increased odds of depressive symptoms
			T2: 1.22 (0.92, 1.62)	0.17	
			T3: 1.58 (1.11, 2.23)	**0.01**	
Akbaraly et al., UK, 2009 [21]	Processed food dietary pattern	Prior depression, age, gender, energy intake	T1: Referent		Increased consumption of processed foods associated with increased odds of depressive symptoms
			T2: 1.44 (1.02, 2.02)	**0.04**	
			T3: 1.83 (1.20, 2.79)	**0.004**	
Akbaraly et al., UK, 2009 [21]	Processed food dietary pattern	Prior depression, age, gender, energy intake, marital status, employment, education, physical activity, smoking	T1: Referent		Increased consumption of processed foods associated with increased odds of depressive symptoms
			T2: 1.41 (1.00, 2.00)	0.05	
			T3: 1.76 (1.14, 2.70)	**0.01**	
Akbaraly et al., UK, 2009 [21]	Processed food dietary pattern	Prior depression, age, gender, energy intake, marital status, employment, education, physical activity, smoking, co-morbidities, use of anti-depressant drugs, cognitive functioning	T1: Referent		Increased consumption of processed foods associated with increased odds of depressive symptoms
			T2: 1.38 (0.98, 1.95)	0.06	
			T3: 1.69 (1.10, 2.60)	**0.02**	
Chatzi et al., Greece, 2011 [15]	Western diet	Age, education, parity, house tenure, depression during previous pregnancies, total energy intake during pregnancy	(Outcome: EPDS)	0.07	No association
			T1: Referent		
			T2: 0.96 (−0.17, 2.00)		
			T3: 1.32 (−0.19, 2.76)		
Chatzi et al., Greece, 2011 [15]	Western diet	Age, education, parity, house tenure, depression during previous pregnancies, total energy intake during pregnancy	(Outcome: symptoms)	0.70	No association
			T1: Referent		
			T2: 1.10 (0.63, 1.93)		
			T3: 1.14 (0.58, 2.26)		
Okubu et al., Japan, 2011 [23]	Western diet	Age, gestation, parity, smoking, change in diet in preceding month, family structure, occupation, family income, education, season, BMI, time of delivery, medical problems during pregnancy, sex and birth weight of baby	Q1: Referent	0.36	No association
			Q2: 0.52 (0.30, 0.93)		
			Q3: 0.71 (0.41, 1.20)		
			Q4: 0.73 (0.42, 1.24)		
Sanchez-Villegas et al., Spain, 2011 [20]	Fast food consumption	Age, sex	Q1: Referent	**0.01**	Increased consumption of fast foods associated with increased odds of self-reported depression
			Q2: 1.00 (0.75, 1.32)		
			Q3: 0.98 (0.73, 1.32)		
			Q4: 1.04 (0.78, 1.39)		
			Q5: 1.45 (1.09, 1.92)		
Sanchez-Villegas et al., Spain, 2011 [20]	Fast food consumption	Age, sex, smoking, physical activity, total energy intake, BMI	Q1: Referent	**0.01**	Increased consumption of fast foods associated with increased odds of self-reported depression
			Q2: 0.99 (0.74, 1.32)		
			Q3: 0.97 (0.72, 1.30)		
			Q4: 1.02 (0.76, 1.38)		
			Q5: 1.40 (1.05, 1.86)		
Sanchez-Villegas et al., Spain, 2011 [20]	Fast food consumption	Age, sex, smoking, physical activity, total energy intake, BMI, consumption of commercial baked goods	Q1: Referent	**0.03**	Increased consumption of fast foods associated with increased odds of self-reported depression
			Q2: 0.99 (0.74, 1.32)		
			Q3: 0.95 (0.70, 1.27)		
			Q4: 1.00 (0.75, 1.35)		
			Q5: 1.36 (1.02, 1.81)		
Sanchez-Villegas et al., Spain, 2011 [20]	Fast food consumption	Age, sex, smoking, physical activity, total energy intake, BMI, consumption of healthy food items	Q1: Referent	**0.02**	Increased consumption of fast foods associated with increased odds of self-reported depression
			Q2: 0.99 (0.74, 1.32)		
			Q3: 0.98 (0.73, 1.32)		
			Q4: 1.03 (0.76, 1.39)		
			Q5: 1.37 (1.02, 1.83)		
Sanchez-Villegas et al., Spain, 2011 [20]	Commercial baked goods consumption	Age, sex	Q1: Referent	0.17	No association
			Q2: 1.38 (1.03, 1.85)		
			Q3: 1.33 (0.99, 1.79)		
			Q4: 1.10 (0.81, 1.49)		
			Q5: 1.40 (1.05, 1.87)		
Sanchez-Villegas et al., Spain, 2011 [20]	Commercial baked goods consumption	Age, sex, smoking, physical activity, total energy intake, BMI	Q1: Referent	0.18	No association
			Q2: 1.44 (1.06, 1.95)		
			Q3: 1.40 (1.01, 1.94)		
			Q4: 1.15 (0.82, 1.61)		
			Q5: 1.43 (1.06, 1.93)		
Sanchez-Villegas et al., Spain, 2011 [20]	Commercial baked goods consumption	Age, sex, smoking, physical activity, total energy intake, BMI, consumption of fast food	Q1: Referent	0.27	No association
			Q2: 1.41 (1.04, 1.93)		
			Q3: 1.37 (0.99, 1.90)		
			Q4: 1.12 (0.79, 1.57)		
			Q5: 1.38 (1.02, 1.87)		
Sanchez-Villegas et al., Spain, 2011 [20]	Commercial baked goods consumption	Age, sex, smoking, physical activity, total energy intake, BMI, consumption of healthy food items	Q1: Referent	0.32	No association
			Q2: 1.42 (1.05, 1.93)		
			Q3: 1.36 (0.98, 1.89)		
			Q4: 1.13 (0.80, 1.58)		
			Q5: 1.37 (1.01, 1.85)		
*Cross-sectional*					
Liu et al., China, 2007 [30]	Fast food	Sex, current year of College study, city, weight, smoking, alcohol	T1: Referent	NS*	
			T2: 0.89 (0.23, 3.46)	**<0.05**	Decreased consumption of fast food associated with reduced odds of depressive symptoms
			T3: 0.40 (0.12, 1.37)		
Liu et al., China, 2007 [30]	Ready to eat food	Sex, current year of College study, city, weight, smoking, alcohol	T1: Referent	NS* **<0.0001**	Decreased consumption of ready to eat food associated with reduced odds of depressive symptoms
			T2: 0.96 (0.77, 1.18)		
			T3: 0.70 (0.57, 0.86)		
Liu et al., China, 2007 [30]	Snack food	Sex, current year of College study, city, weight, smoking, alcohol	*	NS*	Decreased consumption of snack food associated with reduced odds of depressive symptoms
Samieri et al., France, 2008 [25]	Females: Pizza, sandwich	Age, education, income, marital status	Females: 0.21 (−0.11, 0.53) ^**†**^	0.19	No association
Samieri et al., France, 2008 [25]	Biscuits and snacking	Age, education, income, marital status	Males: −0.06 (−0.35, 0.23) ^**†**^	0.70	No association
			Females: 0.13 (−0.07, 0.33) ^**†**^	0.19	No association
Samieri et al.,France, 2008 [25]	Females: Charcuterie, starchy foods^ψ^	Age, education, income, marital status	Females: −0.15 (−0.32, 0.02) ^**†**^	0.07	No association
Jeffery et al., USA, 2009 [19]	High calorie sweet diet	BMI, energy intake	0.012 (*) ^α^	**<0.01**	Decreased consumption of high calorie sweet foods associated with lower mean depressive symptom scores
Jeffery et al., USA, 2009 [19]	High calorie non-sweet diet	BMI, energy intake	−0.018 (*) ^α^	**<0.01**	Decreased consumption of high calorie non-sweet foods associated with lower mean depressive symptom scores
Mikolajczyk et al., Europe, 2009 [38]	Fast food	Country	Males: 1.85 (*)^**†**^	**0.02**	Increased consumption of fast foods associated with greater mean depressive symptom scores for men
			Females 0.34 (*)^**†**^	0.57	No association
Jacka et al., Australia, 2010 [7]	Western dietary pattern	Age, socioeconomic status, education, physical activity, smoking, alcohol, energy intake	C1: Referent	NS*	No association
			C2: 1.52 (0.96, 2.41)		
Nanri et al., Japan, 2010 [22]	Westernized breakfast pattern	Age, sex, workplace	T1: Referent	0.43	No association
			T2: 0.99 (0.63, 1.57)		
			T3: 1.21 (0.75, 1.95)		
Nanri et al., Japan, 2010 [22]	Westernized breakfast pattern	Age, sex, workplace, marital status, BMI, job position, physical activity, smoking, co morbidities, total energy intake	T1: Referent	0.34	No association
			T2: 1.02 (0.64, 1.64)		
			T3: 1.27 (0.77, 2.10)		
Nanri et al., Japan, 2010 [22]	Animal food pattern	Age, sex, workplace	T1: Referent	0.94	No association
			T2: 1.43 (0.92, 2.23)		
			T3: 0.99 (0.63, 1.55)		
Nanri et al., Japan, 2010 [22]	Animal food pattern	Age, sex, workplace, marital status, BMI, job position, physical activity, smoking, co morbidities, total energy intake	T1: Referent	0.91	No association
			T2: 1.47 (0.93, 2.32)		
			T3: 0.97 (0.61, 1.55)		
Fowles, Timmerman et al., USA, 2011 [41]	Fast food frequency	Matched for age, sex	T −2.5 (−6.45, 0.71)	**<0.05**	Increased consumption of fast foods associated with higher mean depressive symptom scores
Jacka et al., Norway, 2011 [8]	Western dietary pattern	Age, income, education, physical activity, smoking, alcohol, energy consumption	Males:		
			C1: Referent		
			C2: 0.87 (0.68, 1.11)	0.25	No association
			Females:		
			C1: Referent		
			C2; 1.25 (0.93, 1.68)	0.27	No association

Results presented as Odds Ratio (OR) or Hazards Ratio (HR) and (95% CI), except where indicated by superscripts: ^†^beta regression coefficients (± SE), or ^α^ mean (±SE).

* Data not provided. ^ψ^ The analysis undertaken for male participants by Samieri et al. [25] was based on a food pattern of meat consumption and thus ineligible for inclusion.

**Table 7:** Summary of associations between depression (exposure of interest) and diet, presented by year of publication

**Author, country, year**	**Type of diet (outcome)**	**Adjusted for confounders**	**Results**	**p value**	**Summary of associations**
*Cross-sectional*					
Pagoto et al., USA, 2009 [24]	Healthy Eating	Age, sex, smoking	−2.03 (0.60) ^**†**^	**0.001**	Depressive symptoms associated with reduced likelihood of healthy eating
Beydoun et al., USA, 2009 [32]	Healthy Eating	Age, poverty status, education, marital status, smoking	White males:		
			(CES-D)–0.25 (0.08) ^**†**^	**<0.05**	Depressive symptoms associated with reduced likelihood of healthy eating
			(CES-D ≥16)–3.44 (1.62) ^**†**^	NS*	Depressive symptoms associated with reduced likelihood of healthy eating
			(CES-D ≥20)–2.82 (1.99) ^**†**^	**<0.05**	No association
			White females:		
			(CES-D)–0.19 (0.07) ^**†**^	**<0.05**	Depressive symptoms associated with reduced likelihood of healthy eating
			(CES-D ≥16)–3.45 (1.26) ^**†**^		Depressive symptoms associated with reduced likelihood of healthy eating
			(CES-D ≥20)–3.93 (1.46) ^**†**^		Depressive symptoms associated with reduced likelihood of healthy eating
Beydoun et al., USA, 2009 [32]	Healthy Eating	Age, poverty status, education, marital status, smoking	African American males:		
			(CES-D)–0.03 (0.07) ^**†**^	NS*	No association
			(CES-D ≥16)–0.08 (1.22) ^**†**^	NS*	No association
			(CES-D ≥20)–0.90 (1.52) ^**†**^	NS*	No association
			African American females:		
			(CES-D)–0.10 (0.06) ^**†**^	<0.1	No association
			(CES-D ≥16)–1.24 (1.04) ^**†**^	NS*	No association
			(CES-D ≥20)–1.22 (1.20) ^**†**^	NS*	No association
Beydoun and Wang, USA, 2010 [33]	Healthy Eating	Age, race/ethnicity, marital status, food insecurity, education, poverty income ratio	Males: −3.29 (2.12) ^**†**^	NS*	No association
			Females: −2.63 (1.96) ^**†**^	NS*	No association
Castellanos et al., USA, 2011 [39]	Fat intake	Age, income, education, fruit/vegetable intake, time in USA	−0.23 (0.14) ^**†**^	0.12	No association
Castellanos et al., USA, 2011 [39]	Fruit and Vegetable consumption	Age, income, education, fat consumption, time in USA	−0.30 (0.09) ^**†**^	**<0.05**	Depressive symptoms associated with reduced likelihood of fruit and vegetable consumption
Crawford et al., USA, 2011 [40]	Frequency of fast food consumption	Age, race, marital status, education, household income, BMI, smoking, physical activity, anti-depressant use	C1: Referent	S*	Depressive symptoms associated with greater fast food consumption
			C2: 1.54 (1.06, 2.25)		

* Data not provided, *S* significant.

Results presented as Odds Ratio (OR) or Hazards Ratio (HR) and (95% CI), except where indicated by superscripts: ^†^beta regression coefficients (± SE), or ^α^ mean (±SE).

### Best evidence synthesis

#### Traditional diets and the risk/likelihood of depression

One high-quality cross-sectional study [28] reported no association between a traditional Mediterranean diet and the likelihood of depressive symptoms. As such, according to the criteria for level of evidence, this is considered to be limited evidence.

Similarly, one high-quality cross-sectional study [8] reported no association between increased adherence to a traditional Norwegian diet and a reduced likelihood of depressive symptoms; again resulting in a limited level of evidence.

One high-quality cohort study [23] identified no association between a traditional Japanese diet and the likelihood of depressive symptoms. In contrast, one high-quality cross-sectional study [22] identified that greater adherence to a traditional Japanese diet was associated with a reduced likelihood of depressive symptoms. Therefore, a conflicting level of evidence exists for an association between a Japanese diet and the likelihood of depression.

#### Healthy, low-calorie, or whole food diets, or well-balanced meals and the risk/likelihood of depression

One high-quality cohort study [23] reported no association between a healthy diet and the likelihood of depression; findings similarly observed by two high-quality cross-sectional studies [7,25]. Furthermore, another high-quality cross-sectional study [34] reported an association between a healthy diet and depression for females (although not for males), whilst a further study [8] reported that an association existed for males (although not for females).

In contrast, one high-quality cohort study [15] reported a significant association between adherence to a healthy diet and a reduced likelihood of depression, an association which was similarly reported by three high-quality cross-sectional studies [26,27,29], and also observed in another cross-sectional study [34] (albeit in females only). Another high-quality cohort study [21] also reported a significant association between increased adherence to a whole food diet and the reduced likelihood of depression. A third high-quality cross-sectional study [7] reported a significant association between the consumption of a traditional diet, characterized by fruit, vegetables, lean meats and whole grains, and a reduced likelihood of depression.

Given that two high-quality cohort and four cross-sectional studies reported a healthy diet reduced the likelihood of depression, whilst one high-quality cohort and various cross-sectional studies reported no association, we report a conflicting level of evidence exists.

#### Western or less healthy diets and the risk/likelihood of depression

Three high-quality cohort studies [15,20,23] and four high-quality cross-sectional studies [7,8,22,25] provided no evidence for an association between a Western diet and the likelihood of depression or depressive symptomatology.

Two analyses from high-quality cohort studies [20,21] and one high-quality cross-sectional study [30] reported significant associations between reduced consumption of Western foods/less healthy diets and a decreased likelihood of depressive symptoms.

Given that two high-quality cohorts and one cross-sectional study reported that the consumption of Western foods/ increased the likelihood of depression, whilst three high-quality cohort and four cross-sectional studies reported no association, we report a conflicting level of evidence exists.

#### Depression as a predictor of diet quality

In studies that examined depression as the exposure variable of interest, one high-quality cross-sectional study [24] reported a significant association between depressive symptoms and the reduced likelihood of eating a healthy diet; that association was also reported in a second high-quality cross-sectional study [32] in white males and females, however not for African American males or females. Similarly, another high-quality cross-sectional study [33] found no association between depression and the likelihood of eating a less healthy diet. Given these data, a conflicting level of evidence exists for the association between depression and diet.

## Discussion

This review identified an emerging body of research that examined the association between diet quality and patterns and the likelihood of depression. Of the available literature, we found only limited evidence to support an association between traditional diets (i.e. Mediterranean diet, Norwegian diet) and depression, after applying a best evidence analysis. We also observed a conflicting level of evidence for associations between (i) a traditional Japanese diet and depression, (ii) a healthy diet and depression, (iii) a Western diet and depression, and (iv) depression and the likelihood of eating a less healthy diet.

When investigating possible reasons for these inconsistent findings overall, as well as amongst comparable studies, similar themes emerged. Notwithstanding the robust methodological quality of most of the included studies, a high level of heterogeneity was observed in relation to the measurement of diet quality, depression assessment and study samples. Particularly notable were the heterogeneous definitions of ‘healthy diet’ and the wide variance in the measurement of diet quality and patterns. It is acknowledged that the complexity in measurement is an inherent issue in this field of research, with high levels of measurement error attenuating observable associations, which may help explain the inconsistencies observed. Indeed, this issue precluded us from conducting a meta-analysis.

Similarly, the wide variance in the instruments used to measure depression may have obscured or diluted potentially significant associations between depression and diet quality. Despite a diagnostic interview being considered a more accurate method for classifying depression than self-report methods, only two studies employed a formal diagnostic tool to identify depression [7,33] with the majority of studies capturing depressive symptomatology rather than depression *per se.* However, it should be acknowledged that some of the studies reviewed had a large sample size which would make the use of diagnostic interviews impractical [36].

Alternatively, variance in the key characteristics of the study populations may help explain the inconsistent findings observed. For example, where some studies used population-based cohorts, others comprised participants who may have greater susceptibility to depression, such as those with pre-diabetes [24] and cancer [26]. It is acknowledged that synthesizing data derived from different study populations increases the likelihood of bias which affects the generalizability and comparability of findings. Moreover, it is plausible that physical illnesses or other conditions such as pregnancy may act as confounders in the relationship between diet quality and depression. A final methodological consideration is the variation in statistical modeling techniques and covariates included across studies to analyze the association between diet and depression. For instance, we draw particular attention to energy intake. The inclusion of this covariate by some (e.g. [19-21] but not all [27,29] may be potentially problematic. While some of the observed associations between depression and diet remained with (e.g. [19-21]) or without [27,29] adjustment for energy consumption, there were instances where significant relationships between (western) dietary pattern and depression was explained by this variable [7], an association that is also observed with regards to anxiety [8]. In this instance, the authors hypothesized that, in the relationship between a ‘western’ dietary pattern and mental health, it may likely be the “absolute amount of unhealthy food consumed” more than the “quantity as a proportion of overall diet’ that is of importance. Furthermore, the high correlation between energy intake and western dietary patterns may be problematic [8]. We recommend that future studies investigating the relationship between diet and depression take this into account. More specifically, we recommend that statistical analyses in this area of research employ *a priori* design, where covariates are explicitly identified prior to the undertaking of analyses to ensure methodological rigor.

If, in fact, a true causal association between diet quality and depression exists but is being masked by methodological shortcomings, this is of great clinical and public health significance. Not only is diet a potentially modifiable risk factor which may support population prevention strategies, but dietary improvement could provide a novel therapeutic strategy for those with existing depression. Given that significant numbers of people fail to respond to pharmacological and/or psychological treatments, this is an area of psychiatric research that warrants greater attention.

It should be noted however, that there are inherent limitations to observational studies in regards to determining causality. The majority of studies included in this review were cross-sectional. Whilst cross-sectional study designs do not provide information regarding the directionality of associations, this review is a reflection of the existing evidence base. We therefore recommend the enactment of higher quality studies that are sufficiently powered to determine causality when exploring the relationship between diet and mental health. Any association between diet quality and patterns and mental illness are likely influenced by a large number of inter-related factors. It is plausible that demographic (e.g. socio-economic position), bio-behavioral, genetic, environmental and socio-cultural factors all contribute to the demonstrated associations. Individual analyses undertaken in the studies included in our review all controlled for some key confounding factors in their analysis, most commonly, age and gender. In addition, the large majority of studies adjusted for socioeconomic variables, such as education and income, as well as other lifestyle behaviors that have been shown to confound the diet-depression relationship [43]. Other studies showed energy intake to contribute to the association between depression and diet [7]. Moreover, the relationship between depression and diet is plausibly bi-directional, with individuals with depression more likely to consume poorer quality diets [44]. Two of the studies [20,21] reviewed tested the ‘reverse causality’ hypothesis and excluded this as an explanation; however, this relationship is complex and difficult to explicate using observational data. It was beyond the scope of this study to examine the overall nutritional components of the dietary patterns and draw comparisons with single food items, however, future studies investigating these links are warranted. The authors acknowledge the variance in the definitions of dietary patterns, which may have reduced comparability of the studies included in this review. However, this issue reflects the broader and inherent complexities often confronted in this area of research. A major strength of this review is that this evidence is for the first time, synthesised and analysed to provide an epidemiological evidence base for the association between diet and depression.

It is also important to note that there is a rapidly developing evidence base supporting the role of diet in the genesis of depression in children [45] and adolescents [46-49]. Therefore, it is acknowledged that a greater number of longitudinal studies that explore the role of diet in the development of mental disorders across the lifespan are required. In the context of this review, having data from a greater number of cohort studies may have altered the findings in the best evidence synthesis. Furthermore, greater variation may exist in dietary habits and quality between, rather than within, countries; a factor that plausibly exists for adults, adolescents and children with regards to depression, as has been seen with regards to the seafood consumption in those with bipolar disorder [50].

We acknowledge that grey literature and dissertations regarding these associations may exist. Whilst the exclusion of these sources of literature may result in our review reflecting less than the existing evidential base, it was beyond the scope of this study to systematically ascertain and review grey literature and dissertations. Notwithstanding the limitations of the available literature, this review has several strengths. To our knowledge, this is the first review to systematically explore associations between diet quality and depression. Our findings provide the basis for further inquiry to determine whether a causal relationship exists between diet quality and depression. Given the relative infancy of this area of research, we suggest that the construction of a standard definition for dietary quality and patterns would enhance future work in this area of enquiry. Higher quality cohort studies using more consistent measures of diet quality or dietary patterns to ensure findings are generalisable and comparable are required; the validation of such tools would, in time, further enhance our understanding of these associations. It is acknowledged that social and cultural factors make the examination of diet as a risk factor for depression challenging, as potential relationships may vary over time and in relation to psychological symptomatology, environmental, and/or contextual factors. Therefore, in addition to quality longitudinal studies, clinical trials designed to evaluate dietary intervention as depression prevention and/or management strategy should be conducted in an attempt to clarify this complex relationship. Moreover, studies that investigate biomarkers as mediators of observed relationships between diet quality and depression may help to clarify potentially causal mechanisms.

## Conclusions

In summary, this systematic review provides a critical summary of the current evidence regarding diet quality and depression, a relatively new field of enquiry. To elucidate whether true causal associations exist between diet and depression, further research is urgently required.

## Competing interests

Lana J Williams has received Grant/Research support from Eli Lilly, Pfizer, The University of Melbourne, Deakin University and the NHMRC. Julie A Pasco has received grant support from the NHMRC, the Geelong Region Medical Research Foundation, Barwon Health, Perpetual Trustees, the Dairy Research and Development Corporation, The University of Melbourne, the Ronald Geoffrey Arnott Foundation, ANZ Charitable Trust, Eli Lilly, the American Society for Bone and Mineral Research and Amgen (Europe) GmBH. Felice N Jacka has received Grant/Research support from the Brain and Behaviour Research Institute (formerly NARSAD), the National Health and Medical Research Council (NHMRC), Australian Rotary Health, the Geelong Medical Research Foundation, the Ian Potter Foundation, Eli Lilly and The University of Melbourne and has been a paid speaker for Sanofi-Synthelabo, Janssen Cilag, Servier, Pfizer, Network Nutrition, and Eli Lilly. Michael Berk has received Grant/Research Support from the NIH, Cooperative Research Centre, Simons Autism Foundation, Cancer Council of Victoria, Stanley Medical Research Foundation, MBF, NHMRC, Beyond Blue, Geelong Medical Research Foundation, Bristol Myers Squibb, Eli Lilly, Glaxo SmithKline, Organon, Novartis, Mayne Pharma and Servier, has been a speaker for Astra Zeneca, Bristol Myers Squibb, Eli Lilly, Glaxo SmithKline, Janssen Cilag, Lundbeck, Merck, Pfizer, Sanofi Synthelabo, Servier, Solvayand Wyeth, and served as a consultant to Astra Zeneca, Bristol Myers Squibb, Eli Lilly, Glaxo SmithKline, Janssen Cilag, Lundbeck and Servier. There are no further conflicting interests/disclosures.

## Authors’ contributions

SEQ and FNJ conceived the study. SEQ, SLB and FNJ designed the study and interpreted the data. SEQ and SLB undertook the acquisition of the data, and took primary responsibility for writing the manuscript. All authors assisted with the interpretation of the analysis and critically revised the manuscript, and read and approved the final manuscript.

## Pre-publication history

The pre-publication history for this paper can be accessed here:

http://www.biomedcentral.com/1471-244X/13/175/prepub

## Supplementary Material

Additional file 1 PRISMA 2009 Checklist. Click here for file
